# Regulatory effects of *Trichinella spiralis* serpin-type serine protease inhibitor on endoplasmic reticulum stress and oxidative stress in host intestinal epithelial cells

**DOI:** 10.1186/s13567-024-01334-6

**Published:** 2024-06-14

**Authors:** Jingbo Zhen, Lihao Lin, Zhixin Li, Feng Sun, Yang Han, Qiankun Li, Yuqi Yang, Xueting Liu, Junchen Yu, Qi Zhang, Yixin Lu, Caixia Han

**Affiliations:** Heilongjiang Provincial Key Laboratory of Zoonosis, College of Veterinary Medicine, Northeast Agricultural University, 600 Changjiang Street, Harbin, 150030 China

**Keywords:** *Trichinella spiralis*, serine protease inhibitor, endoplasmic reticulum stress, oxidative stress

## Abstract

Endoplasmic reticulum stress (ERS) and oxidative stress (OS) are adaptive responses of the body to stressor stimulation. Although it has been verified that *Trichinella spiralis* (*T. spiralis*) can induce ERS and OS in the host, their association is still unclear. Therefore, this study explored whether *T. spiralis*-secreted serpin-type serine protease inhibitor (TsAdSPI) is involved in regulating the relationship between ERS and OS in the host intestine. In this study, mice jejunum and porcine small intestinal epithelial cells (IECs) were detected using qPCR, western blotting, immunohistochemistry (IHC), immunofluorescence (IF), and detection kits. The results showed that ERS- and OS-related indexes changed significantly after TsAdSPI stimulation, and Bip was located in IECs, indicating that TsAdSPI could induce ERS and OS in IECs. After the use of an ERS inhibitor, OS-related indexes were inhibited, suggesting that TsAdSPI-induced OS depends on ERS. When the three ERS signalling pathways, ATF6, IRE1, and PERK, were sequentially suppressed, OS was only regulated by the PERK pathway, and the PERK-eif2α-CHOP-ERO1α axis played a key role. Similarly, the expression of ERS-related indexes and the level of intracellular Ca^2+^ were inhibited after adding the OS inhibitor, and the expression of ERS-related indexes decreased significantly after inhibiting calcium transfer. This finding indicated that TsAdSPI-induced OS could affect ERS by promoting Ca^2+^ efflux from the endoplasmic reticulum. The detection of the ERS and OS sequences revealed that OS occurred before ERS. Finally, changes in apoptosis-related indexes were detected, and the results indicated that TsAdSPI-induced ERS and OS could regulate IEC apoptosis. In conclusion, TsAdSPI induced OS after entering IECs, OS promoted ERS by enhancing Ca^2+^ efflux, and ERS subsequently strengthened OS by activating the PERK-eif2α-CHOP-ERO1α axis. ERS and OS induced by TsAdSPI synergistically promoted IEC apoptosis. This study provides a foundation for exploring the invasion mechanism of *T. spiralis* and the pathogenesis of host intestinal dysfunction after invasion.

## Introduction

Trichinellosis is a global food-borne parasitic disease caused by the consumption of raw meat containing infectious larvae of *Trichinella spiralis* (*T. spiralis*), seriously endangering human health and leading to tremendous economic losses in animal husbandry [1]. When people or animals are infected with *T. spiralis*, muscle larvae are released into the stomach, migrate into the duodenum, burrow into the intestinal mucosa and develop into adult worms through four moults [2]. Then, female worms and male worms mate in the small intestine and give birth to newborn larvae. This indicates that intestinal invasion is an important stage of *T. spiralis* infection and determines the occurrence and development of trichinellosis [3]. Therefore, it is exceedingly important to study the physiological or pathological changes in the host intestine after *T. spiralis* invasion.

After invasion by many parasites, including *Schistosoma japonicum*, *Clonorchis sinensis*, *Ascaris*, *T. spiralis*, *Haemonchus contortus*, and *Brugia malayi*, serine protease inhibitors (SPIs) affect the physiological processes of the host, such as inflammation, coagulation, complement activation, fibrinolysis, cell development, and programmed cell death of host [4]. Our previous studies confirmed that *T. spiralis* serpin-type SPI (TsAdSPI) is an important SPI derived from muscle larvae and adult worms that can cause autophagy in small intestinal cells, disrupt the intestinal mucosal barrier, and regulate the host immune response [1, 5]. However, whether it is involved in other physiological processes of the intestine needs further research.

Endoplasmic reticulum stress (ERS) and oxidative stress (OS) are adaptive responses of hosts after being stimulated by stressors such as parasites, bacteria, and viruses. Some studies have shown that viruses can promote the host to form a favourable living environment for self-replication and pathogenicity by regulating the relationship between ERS and OS after infection [6, 7]. Alfredo et al. [8] showed that the host alleviated *hepatitis C virus*-induced damage through the unfolded protein response (UPR) and antioxidant stress, and initiated an immune response to restore cell homeostasis. However, there are no relevant studies on how parasites regulate the association between ERS and OS, although it has been verified that many parasites, including *T. spiralis*, can cause ERS and OS in the host [9–13]. Thus, on the basis of these studies, this study further explored whether TsAdSPI is involved in the regulatory effect of *T. spiralis* on the relationship between host ERS and OS. Our results will provide a reference for the invasion mechanism of *T. spiralis* and the pathogenesis of trichinellosis.

## Materials and methods

### Animals and cells

BALB/c mice aged 6–8 weeks were purchased from Harbin Medical University. The porcine small intestinal epithelial cell line J2 (IPEC) was generated by Liu Renqiang from Harbin Veterinary Research Institute.

### Preparation of protein, agonists, and inhibitors

The pET-30a-TsAdSPI (accession number: EU263307.1) positive expression bacterial solution was purified on a nickel column (Solarbio, China) and renatured. The endotoxin of the recombinant protein was removed using an endotoxin removal kit (Huzhenbio, China). For the dosage and action time of agonists and inhibitors (MedChemExpress, USA), the reagent specifications and related literature were referenced, as shown in Table 1. Due to the absence of a dosage reference for H_2_O_2_ and EN460, H_2_O_2_ at dosages of 0, 50, 100, 200, 500, and 1000 μM and EN460 at dosages of 0, 4, 8, 12, 16, and 20 mM were cocultured with IPEC for 1 h, respectively. Then, a CCK-8 cell activity detection kit (Meilunbio, China) was used to determine the optimal interaction concentration.

**Table 1:** **Treatment dosage and time of agonists and inhibitors**

Reagent	Function	In vivo experiment		In vitro experiment	
		Dosage	Time	Dosage	Time
4-Phenylbutyric acid (4-PBA)	ERS inhibitor	150 mg/kg	Once a day for 7 d	50 µM	1 h
Tunicamycin (Tm)	ERS agonist	1 mg/kg	24 h	1 µg/mL	3 h
*N*-acetyl-l-cysteine (NAC)	OS inhibitor	150 mg/kg	24 h	1 mM	1 h
Hydrogen peroxide (H_2_O_2_)	OS agonist	0.74 mM/kg	24 h		1 h
4-(2-Aminoethyl)-benzenesulfonyl fluoride hydrochloride (AEBSF)	ATF6 inhibitor			200 µM	1 h
MKC3946	IRE1α inhibitor			10 µM	1 h
GSK2606414	PERK inhibitor			5 µM	1 h
EN460	ERO1α inhibitor				1 h
2-Aminoethoxydiphenyl borate (2-APB)	Ca^2+^ release antagonist			1 µM	1 h

### Preparation of the detection kit and fluorescent probe

Malondialdehyde (MDA) (Nanjing Jiancheng Biology, China), reactive oxygen species (ROS) (Meilunbio, China), and superoxide dismutase (SOD) (Nanjing Jiancheng Biology, China) detection kits were used to measure the MDA and ROS content, and the SOD activity. The Fluo-4 AM fluorescent probe (Meilunbio, China) was used to measure the intracellular Ca^2+^ level. An enzyme-labelled instrument (BioTek, USA) was used to quantify the optical density of MDA and SOD, as well as the fluorescence intensity of ROS and intracellular Ca^2+^. Cell apoptosis was detected by an Annexin V-FITC apoptosis detection kit (Meilunbio, China) and flow cytometry (FCM) (Sagecreation, China). The mitochondrial membrane potential was detected by a mitochondrial membrane potential detection kit (Solarbio, China), and the fluorescence intensity was observed using a fluorescence microscope (BioTek, USA).

### Animal experiments

Sixty-nine BALB/c mice were randomly divided into 23 groups, with 3 mice in each group. Fifteen mice were randomly divided into 5 groups, including the control group, 4-PBA group, Tm group, TsAdSPI group, and TsAdSPI + 4-PBA group, to explore the effects of TsAdSPI on ERS and apoptosis in the host intestine by quantitative real-time polymerase chain reaction (qPCR) and western blotting. Moreover, 15 mice were subjected to immunohistochemistry (IHC) experiments. Then, to study the effects of TsAdSPI on OS and apoptosis, 15 mice were randomly divided into the control group, NAC group, H_2_O_2_ group, TsAdSPI group, and TsAdSPI + NAC group. Finally, 24 mice were randomly divided into 8 groups, namely, the control group, 4-PBA group, NAC group, TsAdSPI group, TsAdSPI + 4-PBA group, and TsAdSPI + NAC group, to explore the effects of TsAdSPI-induced ERS on OS and TsAdSPI-induced OS on ERS. The TsAdSPI, agonists, and inhibitors were injected intraperitoneally into the mice. In the TsAdSPI + inhibitor group, TsAdSPI preceded TsAdSPI, and the dose and stimulation time of TsAdSPI were 50 µg and 24 h, respectively. According to the studies of Xu et al. [3] and Zhen et al. [1], jejunal tissues were selected for subsequent experiments.

### Cell experiments

To probe the effects of TsAdSPI on ERS, OS, and apoptosis in IPEC cells, the cells were divided into the PBS group, 4-PBA group, Tm group, NAC group, H_2_O_2_ group, TsAdSPI group, TsAdSPI + 4-PBA group, and TsAdSPI + NAC group. Then, to explore the interaction between ERS and OS induced by TsAdSPI, the cells were divided into the PBS group, 4-PBA group, NAC group, TsAdSPI group, TsAdSPI + 4-PBA group, and TsAdSPI + NAC group. Next, to study the interactions between ERS and OS, the cells were divided into the PBS group, inhibitor group (including AEBSF, MKC3946, GSK2606414, EN460, and 2-APB), TsAdSPI group, and TsAdSPI + inhibitor group. Finally, to study the activation sequence of ERS and OS, the cells were divided into 0, 1, 2, 3, 6, and 9 h groups. In the TsAdSPI + inhibitor group, the IPEC were treated with inhibitor and then stimulated with 10 µg/mL TsAdSPI for 3 h. The stimulation dosage and time of TsAdSPI were described in a previous study [1].

### qPCR

Total RNA was extracted from tissues or cells with a M5 Universal RNA Mini Kit (Mei5bio, China) and reverse transcribed into cDNA using an All-in-One First Strand cDNA Synthesis Kit III (with dsDNase) for qPCR (Seven Biotech, China). qPCR was performed via a Roche LightCycler 480 system (Roche, Switzerland), and the results were calculated using the 2^−∆∆Ct^ method. The primer sequences are shown in Table 2.

**Table 2:** **Primers of the detected genes**

Gene	Sequence (5ʹ-3ʹ)	Access number	Origin
GAPDH	F: GATTCCACCCACGGCAAGTTCC	NM_001206359.1	Swine
	R: AGCACCAGCATCACCCCATTTG		
GAPDH	F: GTGACGTTGACATCCGTAAAGA	NM_007393.5	Mice
	R: GCCGGACTCATCGTACTCC		
Bip	F: ACCACCTACTCGTGCGTTG	XM_001927795.7	Swine
	R: CGTCGAAGACCGTGTTCTCA		
Bip	F: GCATCACGCCGTCGTATGT	NM_001163434.1	Mice
	R: ATTCCAAGTACATCCGATGAG		
ATF6	F: ATTCCTCCACCTCCCTGTCA	XM_021089515.1	Swine
	R: CCCTGAGTTCCTGCTGATAC		
ATF6	F: CGGTCCACAGACTCGTGTTC	NM_001081304.1	Mice
	R: GCTGTCGCCATATAAGGAAAGG		
CHOP	F: CCCCCTGGAAATGAGGAGGA	NM_001144845.1	Swine
	R: GGAGGTGTGTGTGACCTCTG		
CHOP	F: TATCTTGAGCCTAACACGTCG	NM 001290183.1	Mice
	R: CCAGGTTCTCTCTCCTCAGGT		
ERO1α	F: TGCTTCTGCCAGGTTAGTGG	NM_001137627.1	Swine
	R: ACAAGGCTTGACAGCACAGT		
ERO1α	F: TCAAACCCTGCCATTCTGATGA	NM_015774.3	Mice
	R: CAGAGACTCATCCACGGCTC		

### Western blot

Radioimmunoprecipitation solution (Solarbio, China) was added to the jejunum and IPEC to accelerate lysis. After centrifuging the samples, the supernatants were collected and mixed with 5× protein loading buffer (Beyotime, China). The target proteins were separated via sodium dodecyl sulfate‒polyacrylamide gel electrophoresis and transferred to polyvinylidene difluoride membranes (Biotopped, China). The membranes were sealed with 5% skim milk (Meilunbio, China) at 37 °C for 1 h, and incubated with various antibodies such as anti-β-actin (Bioss, China), anti-immunoglobulin heavy chain binding protein (Bip), anti-activating transcription factor 6 (ATF6), anti-inositol requiring enzyme 1 (IRE1), anti-p-IRE1, anti-protein kinase RNA-like endoplasmic reticulum (ER) kinase (PERK), anti-p-PERK, anti-eukaryotic initiation factor2α (eif2α), anti-p-eif2α, anti-C/EBP homologous protein (CHOP), anti-ER oxidoreductase 1 alpha (ERO1α), anti-pro-cysteme aspartate specific protease 3 (Caspase3), anti-cleaved-Caspase3, anti-B-cell lymphoma 2 (Bcl-2), and anti-Bcl-2 associated X protein (Bax) (Wanleibio, China) at 4 °C overnight. Subsequently, the membranes were incubated with goat anti-rabbit IgG/HRP (Bioss, China) at room temperature for 2 h. The target bands were exposed via supersensitive enhanced chemiluminescence (ECL) reagent (Meilunbio, China) and quantified by an imager (Jiapeng Technology, China). The membranes were washed thrice using PBST as soon as the reagents changed.

### Immunohistochemistry

The jejunum tissues of the mice were made into paraffin slices. After dewaxing, the slices were put into citric acid antigen repair solution. H_2_O_2_ (3%) was added to the slices, which were subsequently incubated at room temperature for 15 min. Then, the slices were blocked with 3% bovine serum albumin (Meilunbio, China) at room temperature for 30 min, incubated with anti-Bip at 4 °C overnight, and enzyme-labelled goat anti-rabbit IgG polymer for 1 h at room temperature. Afterward, the slices were stained with 3,3'-diaminobenzidine and hematoxylin (Origeng, China) for 10 min. Finally, the slices were observed under a fluorescence microscope after dehydration and sealing. The slices were washed three times with PBS or PBST as soon as the reagents changed.

### Immunofluorescence

The treated IPEC was fixed in 4% paraformaldehyde (Saiguobio, China) for 30 min, soaked in 0.25% Triton X-100 (Biotopped, China) for 10 min, and sealed with 2% bovine serum albumin for 30 min at room temperature, respectively. Then, the cells were incubated with anti-Bip at 4 ℃ overnight, goat anti-rabbit IgG HL/FITC (Bioss, China) at 37 ℃ for 1 h in the dark, and DAPI (Bioss, China) at room temperature for 5 min. Finally, the cells were observed by fluorescence microscopy. The cells were washed three times with PBS as soon as the reagents were changed.

### Statistical analysis

The data were statistically and visually analysed by GraphPad Prism 8.0 and are presented as the means ± SD. These data represent three independent experiments, and the average values were compared by one-way analysis of variance (ANOVA). Post hoc ANOVA was performed using the Duncan method in SPSS version 26.0. *P* < 0.05 was considered to indicate statistical significance. ImageJ 1.46r was used to quantitatively analyse the Western blot bands and the IF fluorescence intensity. The magnification of all fluorescence images was 4 × PL FL, and the scale was 1000 μm. IHC images were done at a magnification of 200×.

## Results

### Purification and endotoxin removal of TsAdSPI

**Figure 1:**
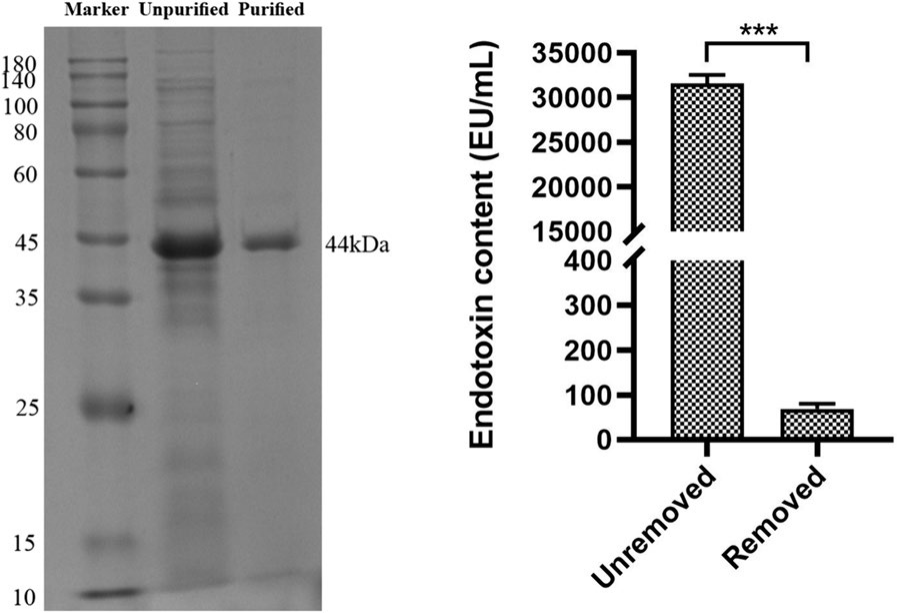
**Purification and endotoxin removal of TsAdSPI.**

TsAdSPI was expressed and purified as a 44 kDa band, and the endotoxin was removed to eliminate its cytotoxicity (Figure 1).

### The optimum dose of H_2_O_2_ and EN460 for interaction with IPEC

**Figure 2:**
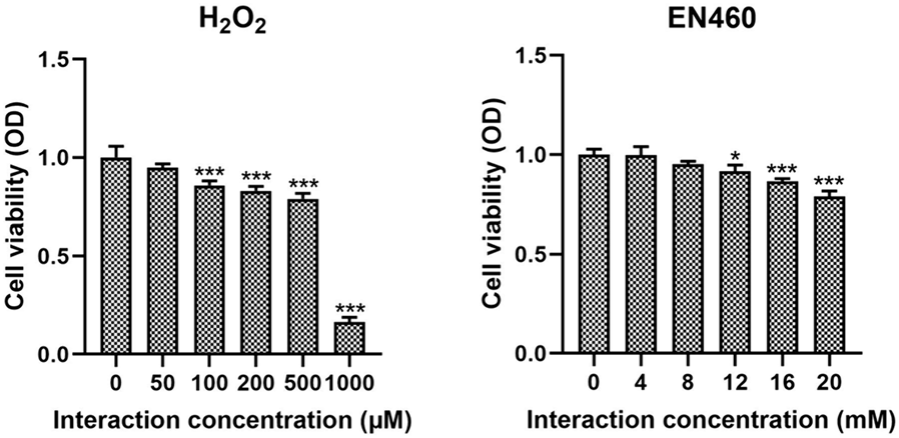
**The optimum doses of H**_**2**_**O**_**2**_** and EN460 for interaction with IPEC.**

The optimum interaction dose between H_2_O_2_, EN460, and IPEC was detected using a CCK-8 cell activity detection kit. The results demonstrated that the optimum concentrations of H_2_O_2_ and EN460 were 100 µM and 16 mM, respectively (Figure 2).

### TsAdSPI induced ERS in IECs

**Figure 3:**
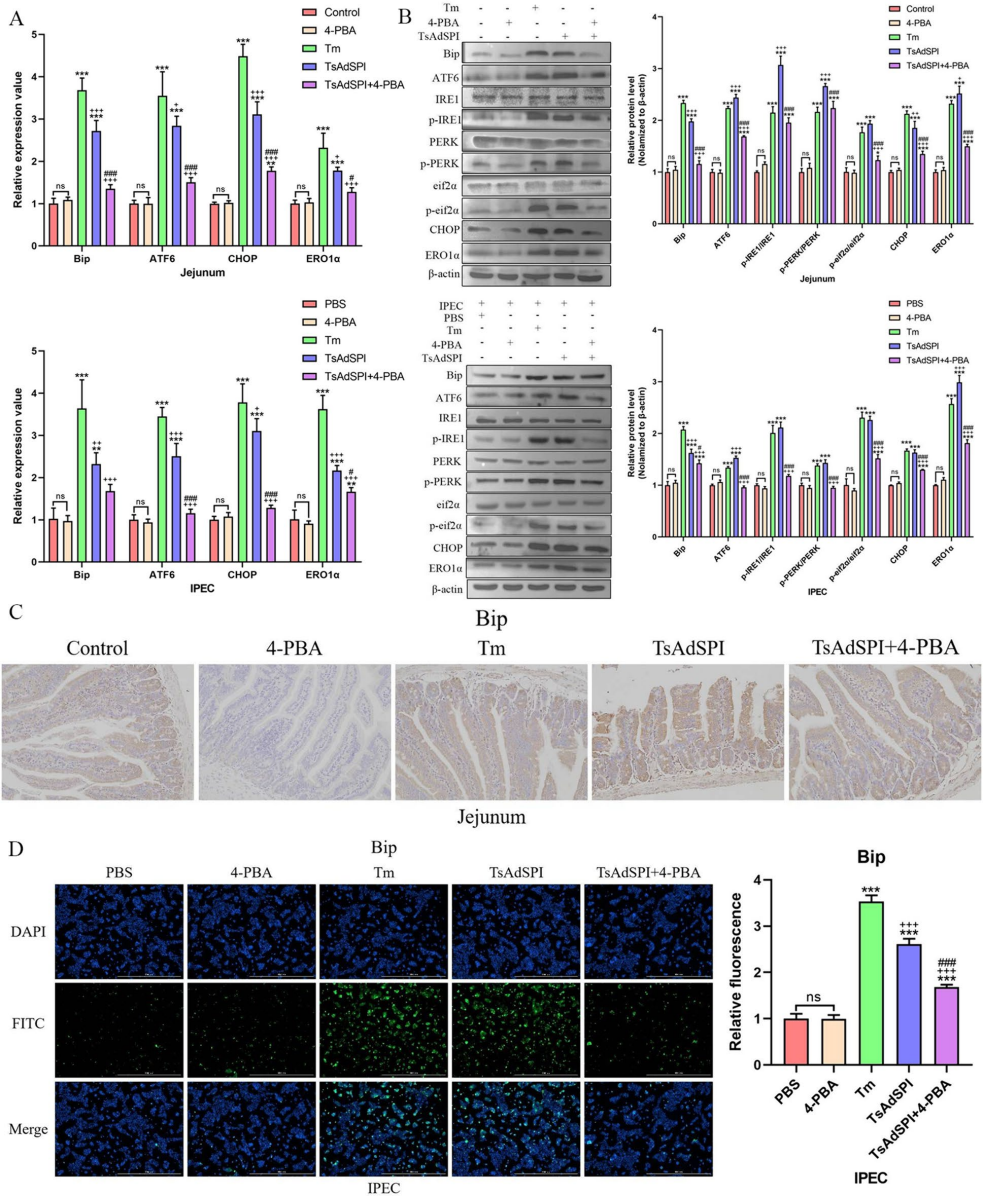
**TsAdSPI-induced ERS in IECs.** qPCR (**A**), Western blot (**B**), IHC (**C**), and IF (**D**) were used to analyse the expression levels of ERS-related genes. * *P* < 0.05, ** *P* < 0.01, *** *P* < 0.001 compared with the control group, PBS group, and 4-PBA group; +*P* < 0.05, ++*P* < 0.01, +++*P* < 0.001 compared with the Tm group; # *P* < 0.05, ## *P* < 0.01, ### *P* < 0.001 compared with the TsAdSPI group.

To study the effect of TsAdSPI on intestinal ERS, in vivo and in vitro experiments were conducted, and the mice and cells were divided into 10 groups, including the control group, PBS group, 4-PBA group, Tm group, TsAdSPI group, and TsAdSPI + 4-PBA group. Changes in ERS-related indexes in the jejunum and IPEC were detected by qPCR, western blotting, IHC, and IF. qPCR revealed that the expression levels of the ERS-promoting genes Bip, ATF6, CHOP, and ERO1α in the control group and PBS group were not significantly different from those in the 4-PBA group (Figure 3A). Compared with those in the above 3 groups, the transcription levels of ERS-related indexes in the Tm group and TsAdSPI group were significantly greater (*P* < 0.001, *P* < 0.01). The expression of ERS-related indexes in the TsAdSPI + 4-PBA group was inhibited in contrast with that in the TsAdSPI group. The western blot results showed that the expression of the ERS-promoting proteins Bip, ATF6, p-IRE1/IRE1, p-PERK/PERK, p-eif2α/eif2α, CHOP, and ERO1α in the TsAdSPI group was significantly greater than that in the control group, PBS group, and 4-PBA group (*P* < 0.001) (Figure 3B). IHC revealed that Bip was mainly expressed in IECs, as shown in the claybank in Figure 3C. The expression in the TsAdSPI group was the highest. The IF results revealed that the expression level of Bip in the TsAdSPI group was significantly greater than that in the PBS group and 4-PBA group (*P* < 0.001) (Figure 3D). These results indicated that TsAdSPI could induce ERS in IECs.

### TsAdSPI induced OS in IECs

**Figure 4:**
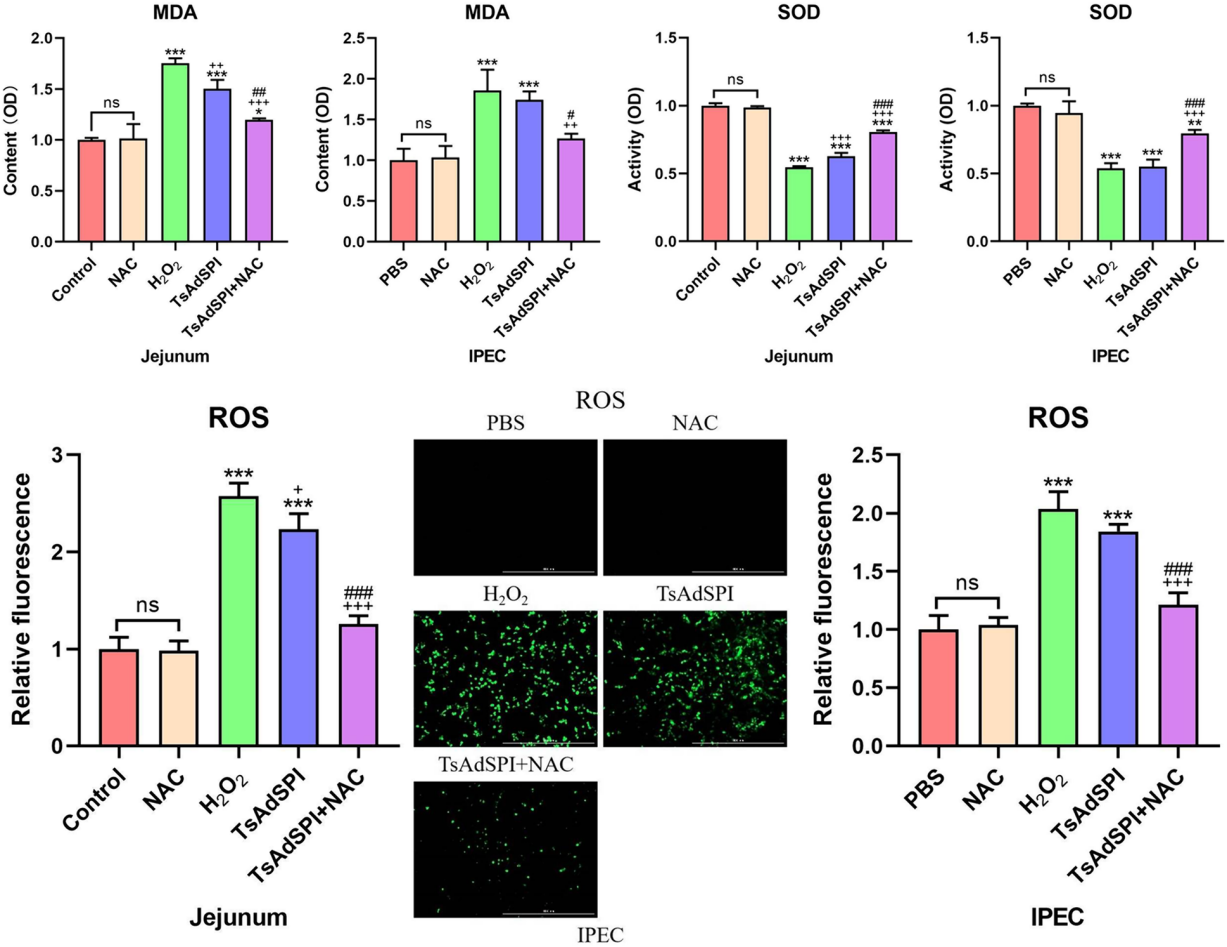
**Identification of TsAdSPI-induced OS in IECs.** MDA, ROS, and SOD assays were used to detect changes in OS-related indexes. * *P* < 0.05, ** *P* < 0.01, *** *P* < 0.001 compared with the control group, PBS group, and NAC group; +*P* < 0.05, ++*P* < 0.01, +++*P* < 0.001 compared with the H_2_O_2_ group; # *P* < 0.05, ## *P* < 0.01, ### *P* < 0.001 compared with the TsAdSPI group.

To investigate the effect of TsAdSPI on intestinal OS, the mice and cells were divided into 10 groups, including the control group, PBS group, NAC group, H_2_O_2_ group, TsAdSPI group, and TsAdSPI + NAC group. MDA, ROS, and SOD assay kits were used to detect changes in OS-related indexes in the jejunum and IPEC. The results showed that there were no significant differences in OS-related indexes between the NAC group and the control group or PBS group. Figure 4 shows that the content of the oxidation index MDA significantly increased in the H_2_O_2_ group and TsAdSPI group in contrast with the above 3 groups (*P* < 0.001). The activity of the antioxidant index SOD was opposite to that of MDA. The fluorescence results of the oxidation indexes ROS showed that TsAdSPI caused a significant increase in ROS content (*P* < 0.001). These results indicated that TsAdSPI could induce OS in IECs.

### TsAdSPI-induced ERS enhanced OS by activating the PERK-eif2α-CHOP-ERO1α axis

**Figure 5:**
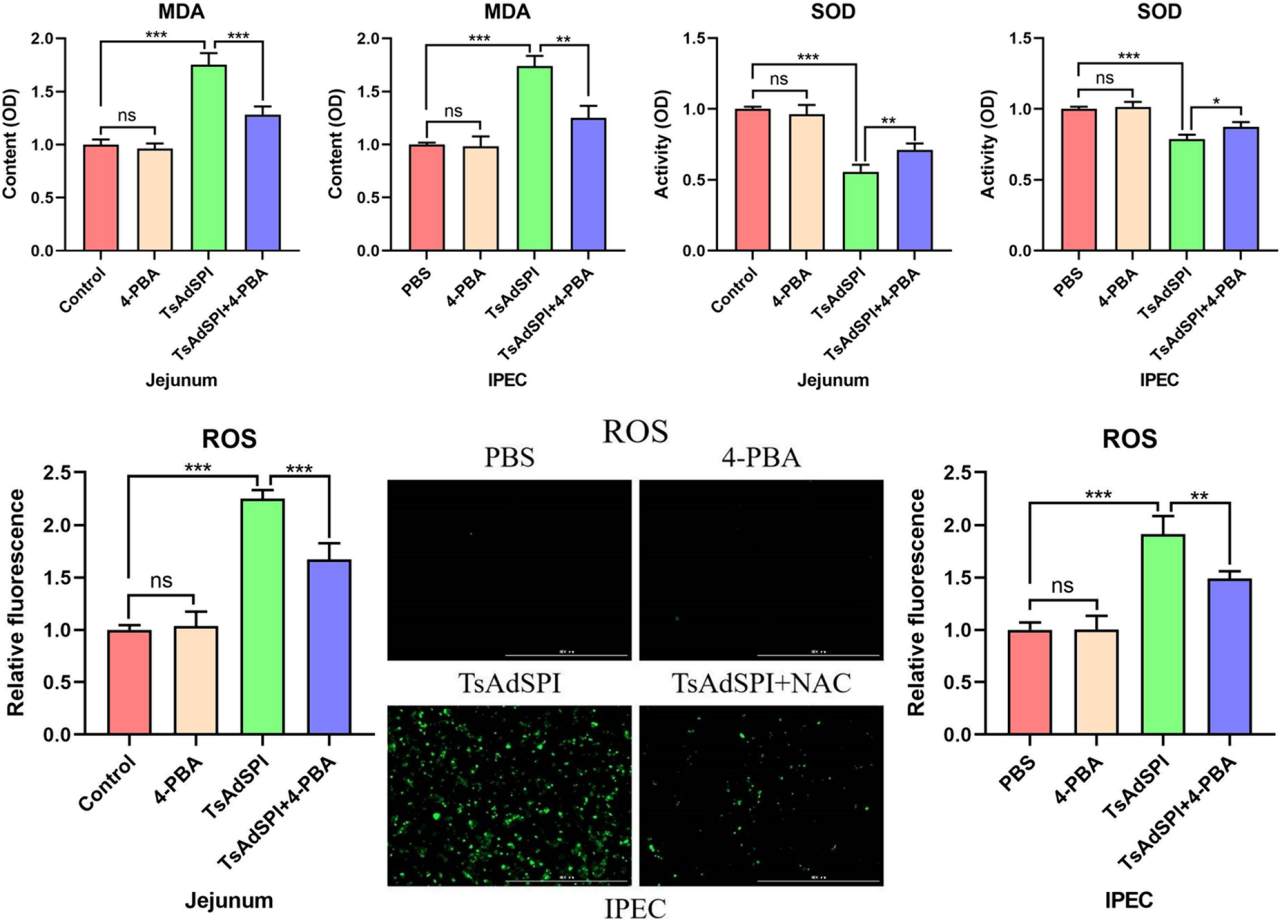
**Identification that TsAdSPI-induced ERS enhanced OS**.

To explore whether TsAdSPI-induced ERS affects OS in IECs, the mice and cells were divided into 8 groups, namely, the control group, PBS group, 4-PBA group, TsAdSPI group, and TsAdSPI + 4-PBA group, and OS-related indexes were detected in the jejunum and IPEC. The results showed that the content of MDA and ROS in the TsAdSPI group increased significantly and the activity of SOD decreased significantly compared with those in the control group and PBS group (*P* < 0.001) (Figure 5). However, after adding the ERS inhibitor 4-PBA, the content of MDA and ROS in the TsAdSPI + 4-PBA group was significantly decreased (*P* < 0.001, *P* < 0.01), and the activity of SOD was considerably increased compared with that in the TsAdSPI group (*P* < 0.01, *P* < 0.05). This finding indicated that TsAdSPI-induced OS depended on ERS and that ERS could increase OS.

**Figure 6:**
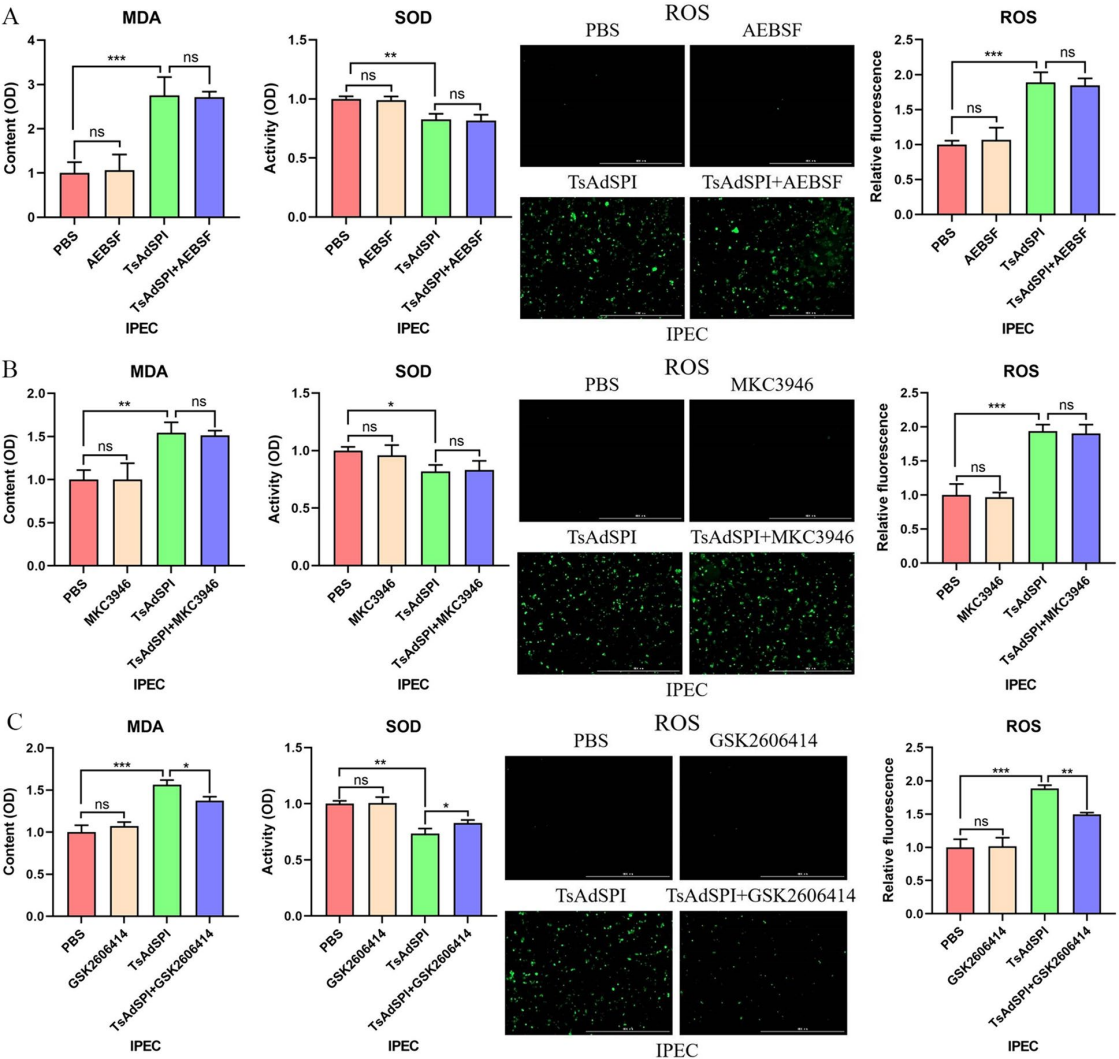
**TsAdSPI-induced ERS enhanced OS by activating the PERK signalling pathway.** Detection kits for MDA, ROS, and SOD were used to analyse the changes in OS-related indexes after inhibiting ATF6 (**A**), IRE1 (**B**), and PERK (**C**).

To further study the ways in which TsAdSPI-induced ERS increased OS, the key proteins ATF6, IRE1 and PERK of the three ERS signalling pathways were inhibited in IPEC. The results showed that OS-related indexes in the TsAdSPI + AEBSF group and TsAdSPI + MKC3946 group were not significantly different from those in the TsAdSPI group after treatment with the ATF6 inhibitor AEBSF and the IRE1 inhibitor MKC3946 (Figures 6A and B). Only after the addition of the PERK inhibitor GSK2606414 did the OS-related indexes of the TsAdSPI + GSK2606414 group change significantly compared with those of the TsAdSPI group (*P* < 0.01, *P* < 0.05) (Figure 6C). These results suggested that TsAdSPI promoted OS by activating the PERK signalling pathway.

**Figure 7:**
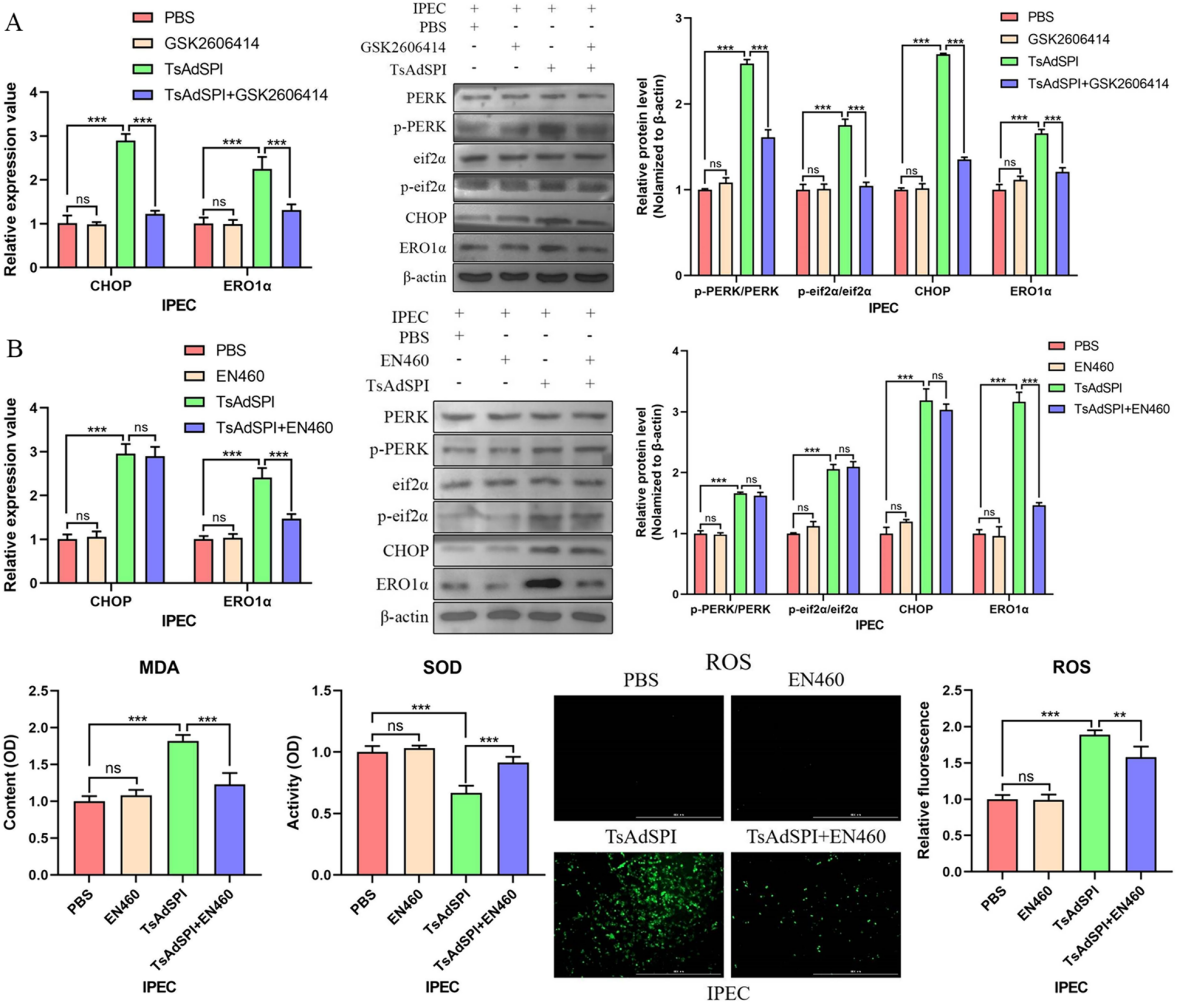
**TsAdSPI-induced ERS enhanced OS by activating the PERK-eif2α-CHOP-ERO1α axis.** qPCR and Western blot analyses of the expression levels of ERS-related indexes after inhibiting PERK (**A**). qPCR, Western blot, and detection kits were used to analyse the changes in ERS- and OS-related indexes after inhibiting ERO1α (**B**).

Subsequently, further in-depth exploration was conducted, and the expression levels of the downstream PERK genes and proteins eif2α, CHOP, and ERO1α in the IPEC were detected via qPCR and western blotting. The results showed that TsAdSPI considerably increased the transcription levels of CHOP and ERO1α and the expression levels of p-PERK/PERK, p-eif2α/eif2α, CHOP, and ERO1α in comparison to those in the PBS group (*P* < 0.001) (Figure 7A). The expression of these genes and proteins was significantly lower in the TsAdSPI + GSK2606414 group than in the TsAdSPI group after the addition of the PERK inhibitor GSK2606414 (*P* < 0.001). After the addition of the ERO1α inhibitor EN460, the expression level of ERO1α in the TsAdSPI + EN460 group decreased significantly (*P* < 0.001), and the expression of ERO1α’s upstream indexes did not change significantly compared with those in the TsAdSPI group (Figure 7B). Consequently, we speculated that TsAdSPI-induced ERS might enhance OS by activating the PERK-eif2α-CHOP-ERO1α axis. The detection results of OS-related indexes confirmed the above conjecture. The contents of MAD and ROS were significantly reduced in the TsAdSPI + EN460 group, while the activity of SOD was significantly upregulated compared with that in the TsAdSPI group (*P* < 0.001, *P* < 0.01).

### TsAdSPI-induced OS enhanced ERS by boosting Ca^2+^ efflux

**Figure 8:**
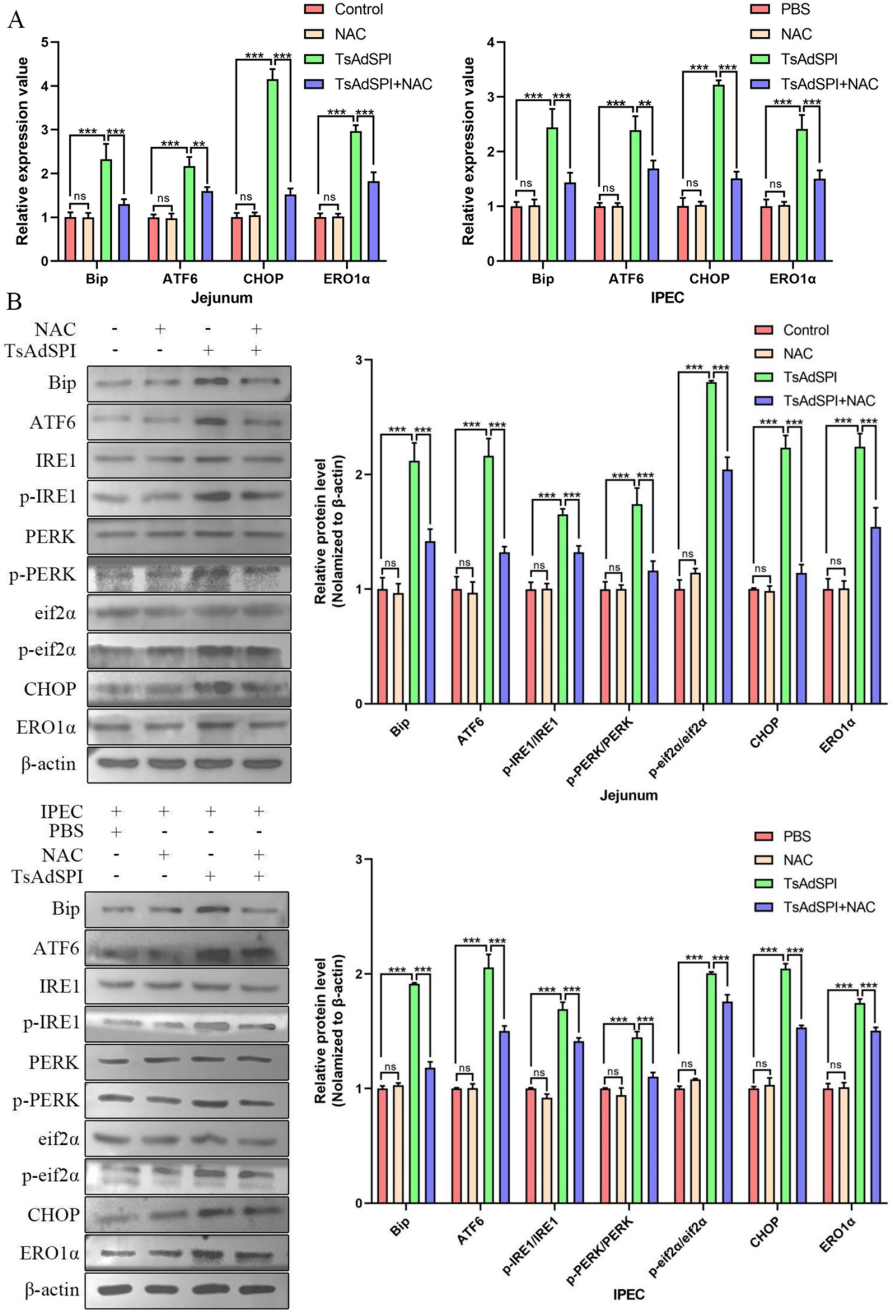
**TsAdSPI-induced OS enhanced ERS.** qPCR (**A**) and Western blot (**B**) analyses of the expression levels of ERS-related indexes.

The above results showed that TsAdSPI-induced OS was partially dependent on ERS, but it remains unclear whether TsAdSPI-induced OS is also dependent on ERS. Therefore, the mice and cells were divided into 8 groups, namely, the control group, PBS group, NAC group, TsAdSPI group, and TsAdSPI + NAC group, and the expression of ERS-related indexes was measured via qPCR and western blotting. qPCR results showed that TsAdSPI significantly increased the transcription levels of Bip, ATF6, CHOP, and ERO1α compared with those in the control group and PBS group (*P* < 0.001) (Figure 8A). Nevertheless, the transcription of these genes was inhibited in the TsAdSPI + NAC group compared with the TsAdSPI group after the addition of the OS inhibitor NAC (*P* < 0.001, *P* < 0.01). The Western blot results displayed a trend similar to that of the qPCR results. The relative expression levels of Bip, ATF6, p-IRE1/IRE1, p-PERK/PERK, p-eif2α/ef2α, CHOP, and ERO1α in the TsAdSPI + NAC group were significantly lower than those in the TsAdSPI group (*P* < 0.001) (Figure 8B). These results demonstrated that the increase in the TsAdSPI-induced ERS level depended on the increase in the OS level, and there was positive feedback between them.

**Figure 9:**
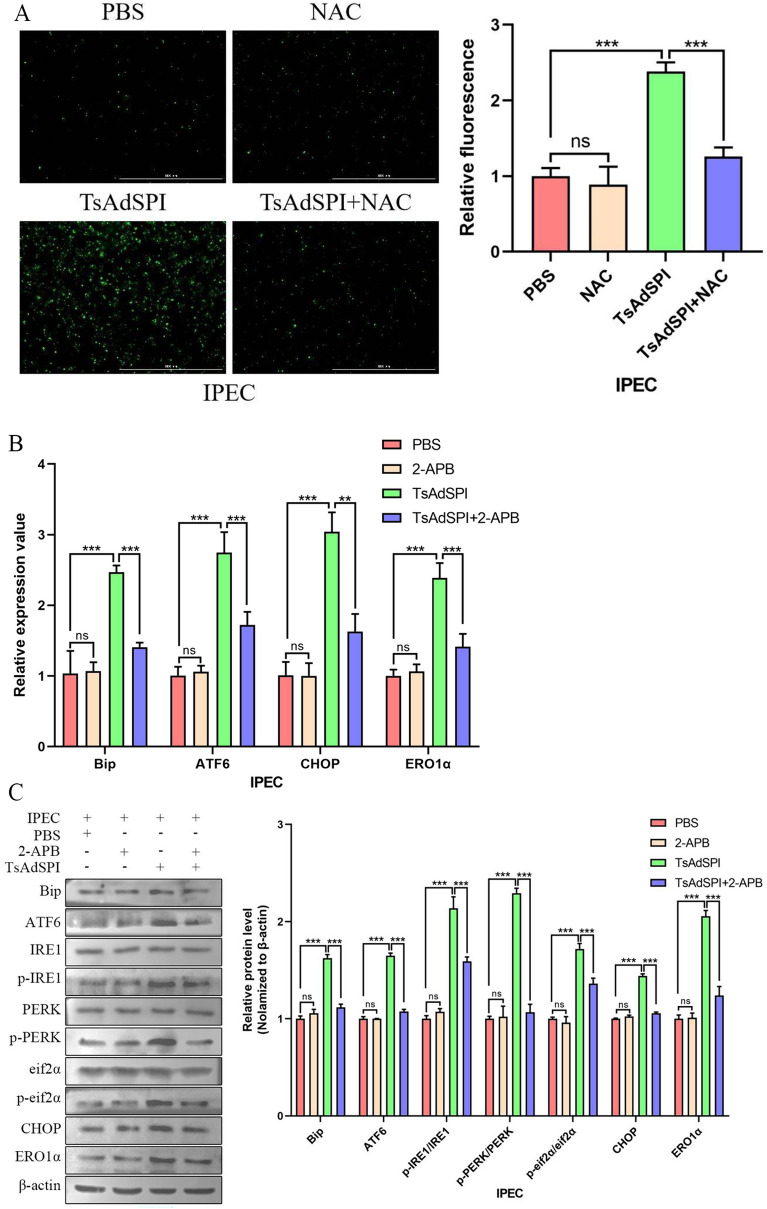
**TsAdSPI-induced OS enhanced ERS by boosting Ca**^**2+**^** efflux.** The fluo-4 AM fluorescent probe (**A**) was used to analyse the Ca^2+^ level in IPEC cells, and qPCR (**B**) and Western blot (**C**) were used to analyse the expression levels of ERS-related indexes.

To further study how TsAdSPI-induced OS promoted ERS, the Ca^2+^ level in the IPEC was detected using the Fluo-4 AM fluorescent probe. Figure 9A shows that the concentration of intracellular Ca^2+^ was significantly greater in the TsAdSPI group than in the PBS group (*P* < 0.001). However, the Ca^2+^ concentration in the TsAdSPI + NAC group was significantly lower than that in the TsAdSPI group, indicating that the increase in Ca^2+^ level induced by TsAdSPI was dependent on OS. Subsequently, the Ca^2+^ release antagonist 2-APB was used to further explore the effects of TsAdSPI-induced OS on Ca^2+ ^and ERS in IPEC in depth. The qPCR and Western blot results showed that the expression levels of ERS-related genes and proteins in the TsAdSPI + 2-APB group were lower than those in the TsAdSPI group after treatment with 2-APB (*P* < 0.001, *P* < 0.01) (Figures 9B and C). Accordingly, we hypothesized that TsAdSPI-induced OS increases ERS by inducing Ca^2+^ efflux from the ER.

### TsAdSPI-induced OS preceded ERS

**Figure 10:**
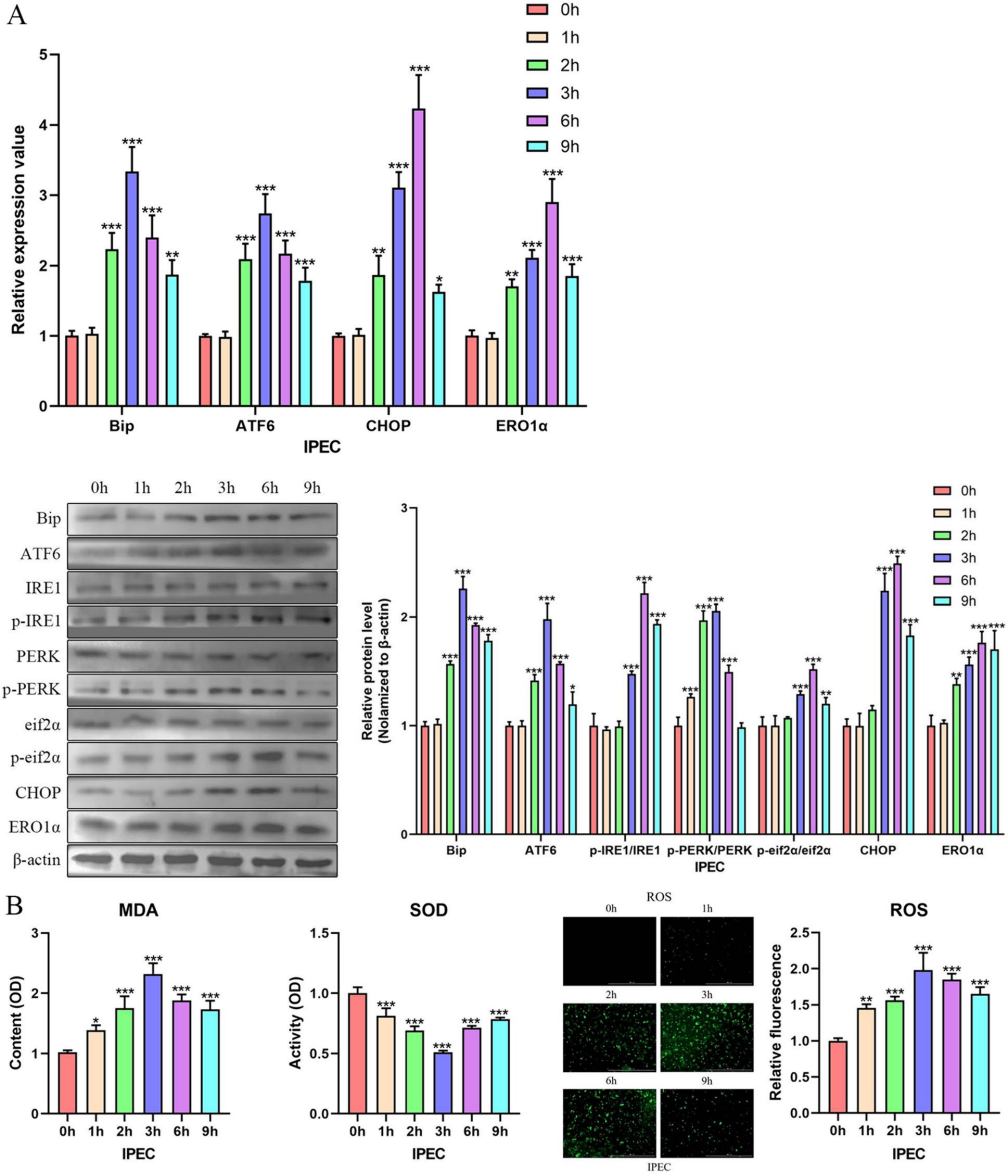
**TsAdSPI-induced OS preceded ERS.** qPCR and Western blotting (**A**) were used to analyse the expression levels of ERS-related indexes. The changes in OS-related indexes were analysed with MDA, ROS, and SOD detection kits (**B**).

To probe the sequence of ERS and OS induced by TsAdSPI, qPCR, western blot, and detection kits were used to observe the changes in ERS- and OS-related indexes 1, 2, 3, 6, and 9 h after IPEC and TsAdSPI were cocultured or not cocultured. Figure 10A shows that there was no significant change in ERS-related indexes after TsAdSPI was used to stimulate IPEC cells for 1 h, but these indexes markedly changed from 2 to 9 h (*P* < 0.001) and peaked at 3 or 6 h. The content of MDA and ROS tended to increase from 1 to 3 h, peaked at 3 h, and then decreased from 3 to 9 h (Figure 10B). The trend of SOD was opposite to that of MDA and ROS and reached its lowest level at 3 h. These results suggested that TsAdSPI-induced OS preceded ERS.

### TsAdSPI promoted IEC apoptosis by enhancing ERS and OS

**Figure 11:**
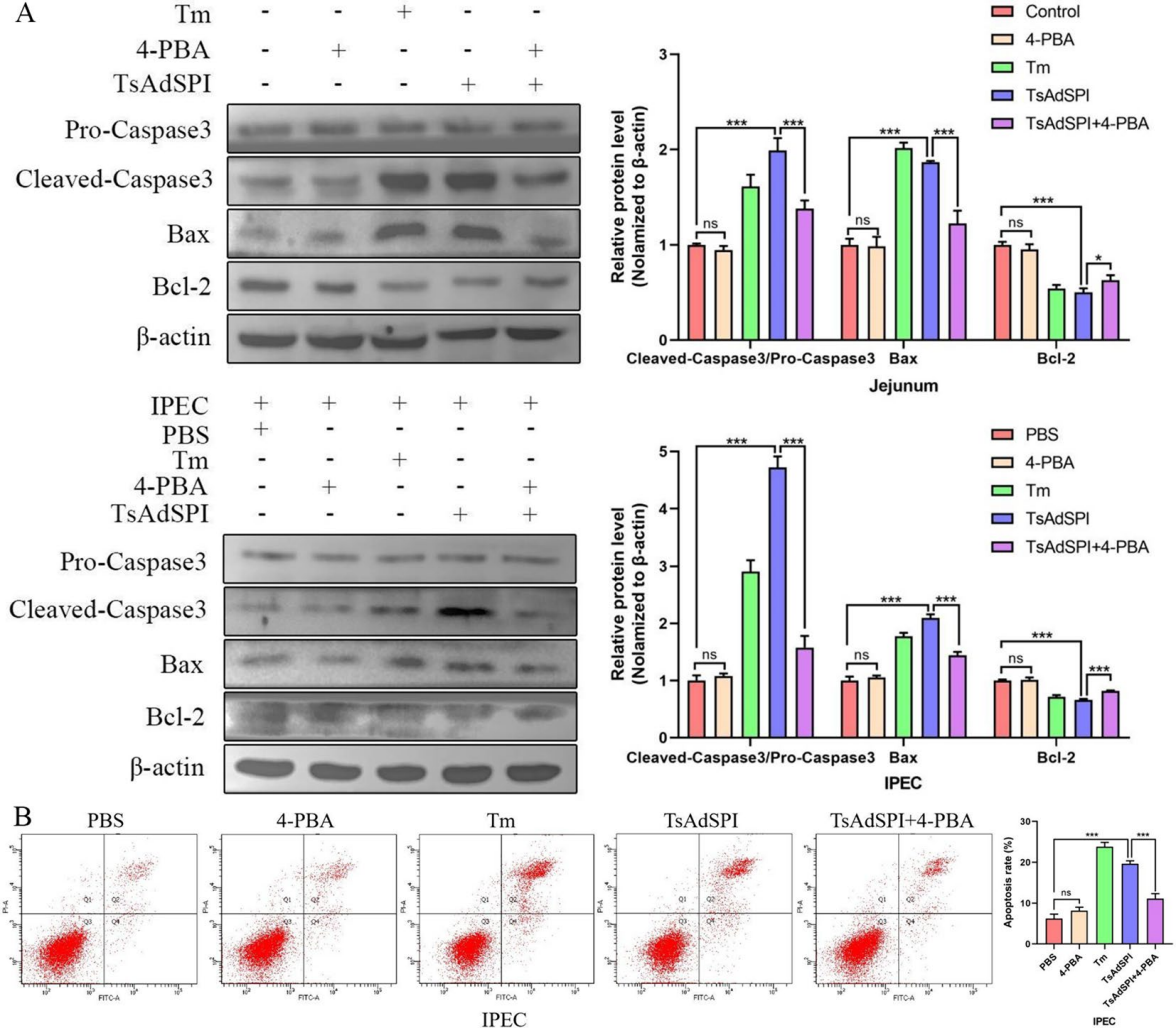
**Identification of TsAdSPI promoted IEC apoptosis by enhancing ERS.** Western blot (**A**) and FCM (**B**) were used to analyse the expression levels of apoptosis-related indexes and changes in the percentage of apoptotic cells.

**Figure 12:**
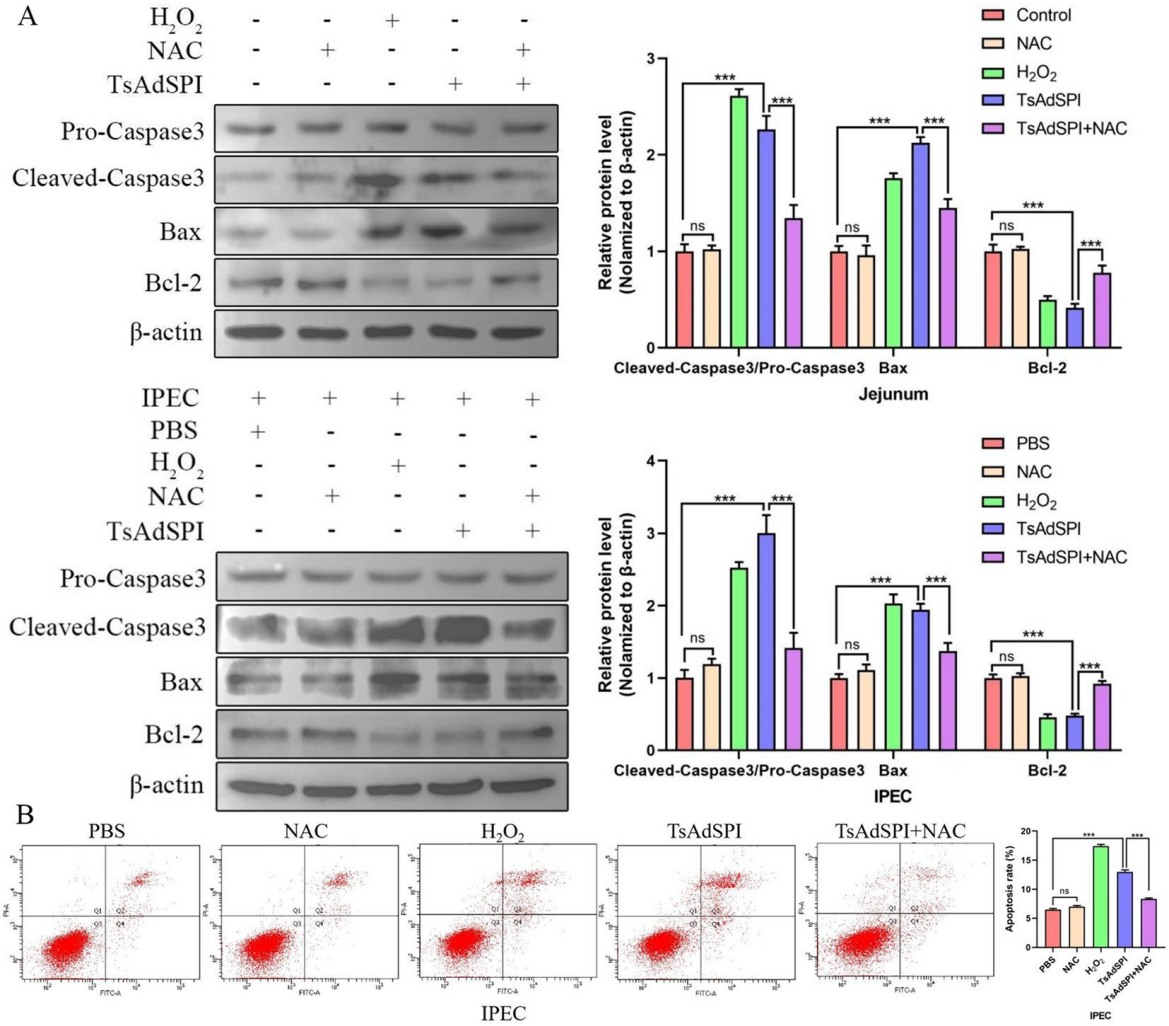
**TsAdSPI promoted IEC apoptosis by enhancing OS.** Western blot (**A**) and FCM (**B**) analyses of the expression levels of apoptosis-related indexes and changes in the percentage of apoptotic cells.

To study the effects of the ERS and OS induced by TsAdSPI on IEC apoptosis, 4-PBA and NAC were used, and the changes in apoptosis-related indexes in the jejunum and IPEC were measured by western blotting and FCM. As shown in Figure 11A, the expression of the apoptosis-promoting proteins cleaved caspase 3/pro-caspase 3 and Bax in the TsAdSPI group was significantly upregulated (*P* < 0.001), and the expression of the apoptosis-inhibiting protein Bcl-2 was dramatically downregulated compared to that in the control group and PBS group (*P* < 0.001). After the addition of 4-PBA, the expression levels of cleaved caspase-3/pro-caspase-3 and Bax in the TsAdSPI + 4-PBA group were significantly decreased, and the expression level of Bcl-2 was significantly increased compared with that in the TsAdSPI group (*P* < 0.001, *P* < 0.05). Furthermore, the rate of apoptosis in the TsAdSPI + 4-PBA group was significantly lower than that in the TsAdSPI group, as shown in Figure 11B (*P* < 0.001). These results showed that TsAdSPI-induced ERS could increase IEC apoptosis. Moreover, the apoptosis-related indexes and the apoptosis rate of the TsAdSPI + NAC group changed significantly compared with those of the TsAdSPI group post-addition of NAC (*P* < 0.001), indicating that TsAdSPI-induced OS could enhance IEC apoptosis (Figure 12).

**Figure 13:**
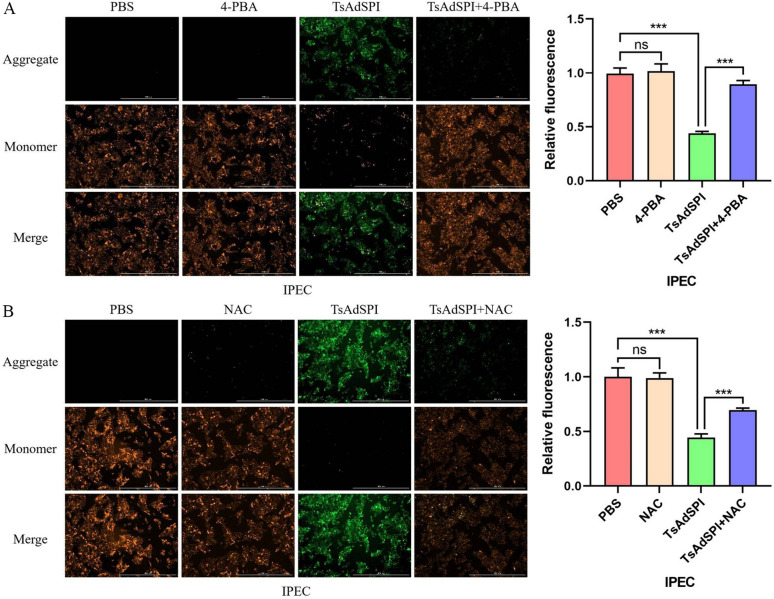
**TTsAdSPI promoted early IEC apoptosis by enhancing ERS and OS.** A mitochondrial membrane potential detection kit was used to analyse the mitochondrial membrane potential of IPEC cells after inhibiting ERS (**A**) and OS (**B**).

To further probe the effects of ERS and OS induced by TsAdSPI on early cell apoptosis in IECs, the mitochondrial membrane potential of IPEC cells was detected using a mitochondrial membrane potential detection kit. Figure 13 shows that TsAdSPI decreased the mitochondrial membrane potential, and the decrease in the mitochondrial membrane potential was alleviated by the addition of 4-PBA and NAC. The results revealed that TsAdSPI promoted early IEC apoptosis by enhancing ERS and OS.

## Discussion

Many studies have shown that the host reacts to defend against the invasion of exogenous pathogens such as parasites, bacteria, and viruses. However, pathogens can benefit their own survival by inducing ERS and OS in host cells and achieve a balance between damaging host cells and reproduction [14, 15]. Therefore, on the basis that *T. spiralis* can induce ERS and OS in the host, this study further explored whether *T. spiralis*-secreted TsAdSPI participates in inducing ERS and OS and how to regulate the relationship between them.

ERS is a protective stress process in which cells respond to misfolding, unfolded protein aggregation, and calcium imbalance in the ER cavity and restore ER homeostasis by activating signalling pathways such as the UPR, the ER overload reaction, and apoptosis [10]. As a receptor and molecular chaperone of ER homeostasis, Bip plays an important role in monitoring UPR aggregation and ER activation. During ER homeostasis, Bip binds to the ER transmembrane proteins ATF6, IRE1, and PERK. When the UPR increases, Bip has a greater affinity for the UPR and dissociates from ATF6, IRE1, and PERK, activating three main signalling pathways of the UPR [16]. In our study, Bip, ATF6, IRE1, and PERK and their downstream proteins eif2α, CHOP, and ERO1α were detected to determine the effect of TsAdSPI on ERS in the jejunum and IPEC. The results of qPCR, Western blot, IHC, and IF showed that these indexes were significantly upregulated after TsAdSPI stimulation, and Bip was located in IECs, which indicated that TsAdSPI could induce ERS in host IECs.

When people or animals are stimulated by stressors, the redox system of the body is imbalanced, resulting in ROS accumulation and triggering OS. An appropriate amount of ROS can help the body eliminate redundant or harmful cells in a timely manner, which is the normal defense mechanism of the body. Nevertheless, when excessive ROS accumulate, cell homeostasis disorders occur, leading to irreversible cell damage and apoptosis [17]. An increase in the oxidation indexes ROS and MDA and a decrease in the antioxidant index SOD are signs of OS occurrence [18]. Through in vivo and in vitro experiments, we found that ROS, MDA, and SOD in IECs exhibited the same changes as those described above after TsAdSPI stimulation, indicating that TsAdSPI can induce OS in IECs.

Previous results have shown that TsAdSPI can enhance ERS and OS in IECs; therefore, whether there is a connection between them and whether TsAdSPI-induced ERS can affect OS remain unclear. To clarify this, an ERS inhibitor was used, and OS-related indexes were detected. When ERS was inhibited, OS was also inhibited, which indicated that TsAdSPI-induced OS depended on ERS and that ERS could enhance OS. Moreover, the key proteins ATF6, IRE1, and PERK in the three ERS pathways were inhibited, and OS was only regulated by PERK, while ATF6 and IRE1 had no regulatory effect on OS. We subsequently inhibited PERK and detected the expression levels of the downstream PERK genes and proteins eif2α, CHOP, and ERO1α. The results showed that the expression of these three indexes was decreased, and the levels of ROS and MDA were also decreased. This indicated that TsAdSPI-induced ERS could promote OS by activating the PERK-eif2α-CHOP-ERO1α axis. Zhou et al. [19] found that *Porcine epidemic diarrhea virus * disrupted the redox homeostasis of the host by manipulating the PERK-CHOP-ERO1α-ROS axis, which was beneficial for self-replication. This confirmed our results to some extent.

Furthermore, we explored the effect of TsAdSPI-induced OS on ERS. The results showed that the expression of ERS-related indexes decreased significantly after treatment with the OS inhibitor, suggesting that TsAdSPI-induced OS depended on ERS and that there was a synergistic relationship between them. Zeeshan et al. [20] reported that calcium is a redox signalling medium that regulates the relationship between ERS and OS. For this reason, we measured the concentration of Ca^2+^ in the IPEC to determine whether TsAdSPI-induced OS boosted ERS by affecting calcium transfer. The results showed that the intracellular Ca^2+^ level decreased after OS was inhibited. After the addition of a Ca^2+^ releasing antagonist, the expression of ERS-related indexes decreased significantly. This revealed that TsAdSPI-induced OS could strengthen ERS by promoting Ca^2+^ efflux.

Subsequently, the sequence of ERS and OS was determined by detecting the changes in ERS- and OS-related indexes after TsAdSPI was used to stimulate IPEC for different durations. The results showed that OS-related indexes changed significantly at 1 h and peaked at 3 h post stimulation, but the expression of ERS-related indexes reached their highest levels at 3 or 6 h, indicating that TsAdSPI-induced OS preceded ERS. Thus, we speculated that the IECs produced OS after TsAdSPI stimulation, which caused a redox imbalance in the ER, resulting in the disturbance of ER function and triggering ERS. Moreover, the occurrence of ERS produces a large amount of ROS and exacerbates OS. ERS and OS showed a relationship of mutual correlation and promotion.

Apoptosis is activated when cells are under continuous or excessive stress. This adaptive regulatory process can be activated by signalling pathways such as the IRE1-Caspase12, PERK-Eif2α-CHOP, ROS-fatty acid synthase ligand-Caspase8, and ROS-apoptotic signal regulated kinase 1 pathways [21, 22]. Some studies have shown that the ERS and OS mediated by parasites can induce the apoptosis of host cells [12, 23]. Hence, we speculated that TsAdSPI was involved in the apoptosis of host cells induced by *T. spiralis*-mediated ERS and OS. The experimental results confirmed the above conjecture: TsAdSPI-induced apoptosis was inhibited after inhibiting ERS and OS. This indicated that TsAdSPI-induced IEC apoptosis depended on ERS and OS and that ERS and OS were closely related to apoptosis.

In conclusion, after TsAdSPI induced OS after entering IECs, OS promoted ERS by enhancing Ca^2+^ efflux from the ER, and ERS subsequently strengthened OS by activating the PERK-eif2α-CHOP-ERO1α axis. The combination of ERS and OS induced by TsAdSPI synergistically promoted IEC apoptosis. These processes may help *T. spiralis* achieve a balance between host cell damage and its own survival and contribute to its parasitism.

## Data Availability

The datasets used or analysed during the current study are available from the corresponding author upon reasonable request.
